# Decrease in Tuberculosis Cases during COVID-19 Pandemic as Reflected by Outpatient Pharmacy Data, United States, 2020 

**DOI:** 10.3201/eid2804.212014

**Published:** 2022-04

**Authors:** Kathryn Winglee, Andrew N. Hill, Adam J. Langer, Julie L. Self

**Affiliations:** Centers for Disease Control and Prevention, Atlanta, Georgia, USA

**Keywords:** COVID-19, tuberculosis and other mycobacteria, coronavirus disease, SARS-CoV-2, severe acute respiratory syndrome coronavirus 2, viruses, respiratory infections, zoonoses, surveillance, prescriptions, SARIMA, United States, bacteria

## Abstract

We analyzed a pharmacy dataset to assess the 20% decline in tuberculosis (TB) cases reported to the US National Tuberculosis Surveillance System (NTSS) during the coronavirus disease pandemic in 2020 compared with the 2016–2019 average. We examined the correlation between TB medication dispensing data to TB case counts in NTSS and used a seasonal autoregressive integrated moving average model to predict expected 2020 counts. Trends in the TB medication data were correlated with trends in NTSS data during 2006–2019. There were fewer prescriptions and cases in 2020 than would be expected on the basis of previous trends. This decrease was particularly large during April–May 2020. These data are consistent with NTSS data, suggesting that underreporting is not occurring but not ruling out underdiagnosis or actual decline. Understanding the mechanisms behind the 2020 decline in reported TB cases will help TB programs better prepare for postpandemic cases.

The coronavirus disease (COVID-19) pandemic has affected many areas of public health, including tuberculosis (TB) prevention and response activities (*1*). TB cases reported to the US National Tuberculosis Surveillance System (NTSS) in 2020 decreased 20% compared with the average number of cases reported during 2016–2019 (*2*). Although some annual decline is expected on the basis of public health investments in TB control and prevention, TB incidence decreased an average of only 2%–3% annually during the previous 10 years (*3*). A decline of nearly 20% raises concern that TB cases are being left undetected or unreported to public health agencies. A sharp decline in TB incidence in 2020 is possible, potentially because of control efforts undertaken to combat the COVID-19 pandemic or reduced immigration, leading to fewer cases among persons newly arriving in the United States from regions with higher TB incidence. We therefore sought to determine the extent to which this decline is actual, a surveillance artifact caused by underreporting, or representative of delayed or missed TB diagnoses. Understanding the underlying cause will help TB programs better allocate resources and prepare for TB cases after the pandemic. Analysis of TB-related trends in data sources unlikely to be affected by public health disruptions is a critical way to evaluate the mechanisms behind the reported decline.

One such source is the IQVIA (https://www.iqvia.com) prescription dataset, which captures >88% of all outpatient prescription activity in the United States, including retail, mail, and long-term care channels. These data have been used in public health to answer a variety of questions, including estimating costs of HIV preexposure prophylaxis (PrEP) (*4*); analyzing the demographics of persons who have been prescribed PrEP (*5*); identifying opioid prescription patterns (*6*–*8*); and assessing naloxone, antibiotic, and hydroxychloroquine prescriptions (*9*–*12*). Pharmacy data are particularly valuable for TB disease because of the unique drug regimens used to treat TB. Initial treatment for newly diagnosed drug-susceptible TB disease typically consists of 4 drugs: isoniazid, rifampin, ethambutol, and pyrazinamide (*13*). All 4 drugs are taken in the first 2 months. If drug susceptibility testing results do not demonstrate resistance to isoniazid or rifampin, this intensive phase is typically followed by a continuation phase consisting of just isoniazid and rifampin for an additional 2–4 months or longer, depending on response to treatment. Rifampin is used to treat multiple diseases, including TB, *Neisseria meningitidis, Haemophilus influenzae*, leprosy, and endocarditis. Rifampin and isoniazid (alone or in combination) are also used to treat latent TB infection (LTBI). Ethambutol is used to treat TB and nontuberculous mycobacteria. Isoniazid is used to treat TB disease and LTBI and is rarely used for other diseases. Pyrazinamide is only used to treat TB disease. Thus, we focused on individual isoniazid and pyrazinamide prescriptions, because these prescriptions should generally indicate TB treatment even in the absence of information on concurrent prescriptions (*14*).

Although most US TB cases are treated in public health clinics that dispense their own medication, some cases are treated by private providers or by clinics that have their TB medications filled by retail pharmacies. Therefore, although it would not be practical to determine overall US TB disease incidence on the basis of outpatient pharmacy dispensing data, assessing the trend in dispensing of TB drugs is possible. We first determined whether the trends in IQVIA’s TB medication prescription data correlated with trends observed in NTSS data before the pandemic (pre-2020). Once this correlation was established, we compared changes in IQVIA TB prescription data with changes in TB cases reported to NTSS in 2020 to assess potential underreporting of TB cases to public health.

## Methods

### IQVIA Metrics

We used 5 different IQVIA databases in this analysis: National Prescription Audit (NPA), NPA Extended Insights, NPA New to Brand (NTB), NPA Regional, and Total Patient Tracker (TPT) (Appendix). Data used in all pyrazinamide and isoniazid analyses were accessed March 8–11, 2021, so the most recent prescriptions available in all databases were from February 2021. We also selected azithromycin as a control antibiotic to look at the specificity of the analyses to TB data and generated azithromycin data on June 15, 2021. 

### NTSS Metrics

We used the provisional 2020 NTSS data frozen in February 2021 and reported in March 2021 (*2*). We removed all TB cases reported by US territories and freely associated states, leaving just cases reported by the 50 US states and the District of Columbia. Cases were aggregated either by treatment start date or case date. Case date was defined as the earliest of treatment start date, drug susceptibility testing date, and report date. If a case was missing the date used for aggregation (7.8% for treatment start date and 1.7% for case date), we excluded it from the analysis.

For comparison to the IQVIA dataset, we tested both including all cases and removing cases with drug resistance (which are unlikely to have been prescribed the drugs of interest). Removing cases with resistance meant removing all cases in which resistance to the drugs of interest was reported on either the first or last isolate for which testing was performed, as well as all cases for which the drug was not part of the initial treatment regimen and was not taken for >2 weeks. For isoniazid, we also removed cases that were rifampin resistant, because rifampin-resistant cases are generally not treated with isoniazid. This activity was reviewed by Centers for Disease Control and Prevention and was conducted consistent with applicable federal law and organization policy (e.g., 45 C.F.R. part 46, 21 C.F.R. part 56; 42 U.S.C. §241(d); 5 U.S.C. §552a; 44 U.S.C. §3501 et seq).

### Correlation Analyses

We made pairwise comparisons at the national level for all IQVIA databases (except NPA Regional) and metrics for both isoniazid and pyrazinamide against NTSS case counts. NTSS case counts either included all patients or removed cases with resistance and were aggregated either by case date or treatment start date. We tested aggregating the data at the month and quarter level. We used a Pearson correlation coefficient (*r*) for the aggregated dates available in both datasets to identify the combination (IQVIA database, NTSS aggregation, and month/quarter timeframe) with the strongest correlation (highest *r*), which was used in subsequent analyses. For visualization, we generated a linear model with IQVIA data as the outcome and NTSS data as the only covariate. We plotted model estimates and 95% prediction intervals with the data.

We conducted similar analyses at the state level by using NPA Regional (data not shown) to explore correlations at smaller geographic levels. On the basis of this analysis, we compared NTSS case counts with a treatment start date in 2019 or 2020 with resistant cases removed to IQVIA isoniazid or pyrazinamide New Prescription (NRx) counts in 2019 or 2020.

### Modeling

Seasonal autoregressive integrated moving average (SARIMA) models used to predict 2020 patient counts were built on data from January 2006–December 2019 using all NTSS cases (aggregated by treatment start date month) or TPT projected patient counts for isoniazid and pyrazinamide. SARIMA models have been used previously to explore TB case counts in the United States (*15*). We fit models by using the auto.arima function with seasonal models from the R package fpp2 (*16*,*17*). We also generated a linear model between the 2 datasets, which we used to predict IQVIA 2020 counts from NTSS 2020 counts and compare those with actual 2020 IQVIA counts (Appendix). We performed all analyses using R version 4.0.2 (*18*).

## Results

### Correlation between IQVIA and NTSS Prepandemic

**Figure 1:**
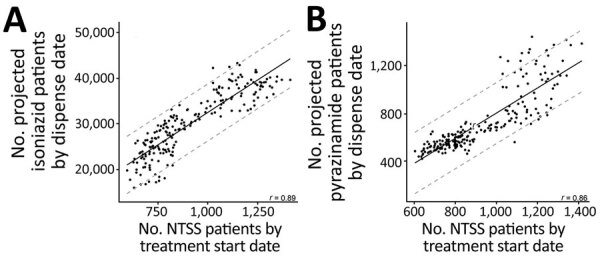
Correlation between National Tuberculosis Surveillance System case counts and IQVIA (https://www.iqvia.com) projected patient counts for isoniazid (A) or pyrazinamide (B) prescriptions, United States, 2006–2019. Horizontal axes show patient counts aggregated by treatment start date, removing patients who had reported resistance to isoniazid or pyrazinamide. Vertical axes show IQVIA projected patient counts aggregated by date drug was dispensed (data are on different scales). Each point represents a month, and all data during 2006–2019 are shown. Solid black lines represent regression fit for a linear model between the 2 databases; dashed gray lines indicate 95% prediction intervals. The Pearson correlation coefficient (*r*) is shown in the lower righthand corner of each plot.

We first examined whether the trends identified in the IQVIA database correlate to trends in NTSS. For both drugs, the strongest correlation occurred when the databases were aggregated by month, NTSS aggregated by treatment start date after removing patients with resistance to the drug of interest and IQVIA aggregated by the TPT projected patient count totals (Appendix Tables 3, 4). This combination had a strong correlation for both drugs (r = 0.89 for isoniazid and 0.86 for pyrazinamide; Figure 1). We therefore used monthly aggregation wherever possible. To confirm the specificity of this relationship, we calculated the correlation between NTSS case counts and IQVIA projected patient counts for azithromycin, an antibiotic not used to treat TB (Appendix Figure 1). This *r* was −0.61, lower in magnitude and opposite in sign compared with TB medications.

We further explored this relationship by comparing additional patient data in both databases. Although the relative proportions of age groups varied, in 2019 and 2020 >50% of patients were >45 years of age in both NTSS and IQVIA (Appendix Figure 2, panel A). NTSS and IQVIA isoniazid had a higher percentage of male than female patients in 2019 and 2020, whereas IQVIA pyrazinamide projected patient counts had a higher proportion of female than male patients. We also compared geographic distributions by using the NPA Regional NRx metric. We found a very high correlation between NTSS patient counts and IQVIA’s NRx for isoniazid (*r* = 0.91) and pyrazinamide (*r* = 0.92) in 2019 and 2020 (*r* = 0.92 and *r* = 0.94; Appendix Figure 3). The correlation between NTSS case counts was weaker but still strong when looking at azithromycin (Appendix Figure 1, panel B).

### Large Decline in Both Databases in 2020

**Figure 2:**
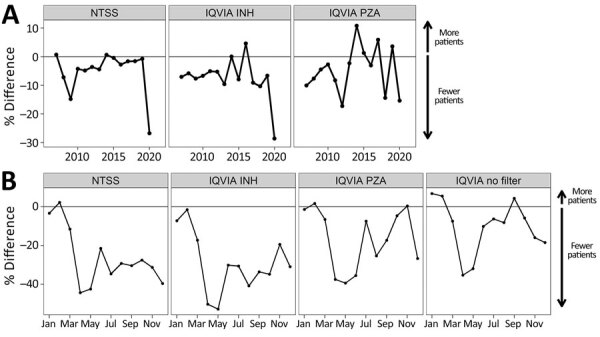
Comparison between 2020 tuberculosis case counts and case counts in previous years, United States. A) Percentage difference in case counts each year compared to previous years. NTSS case counts were aggregated by treatment start date month. A moving annual total ending in December was used for the number of projected patients in the IQVIA (https://www.iqvia.com) dataset prescribed INH or PZA. The horizontal black line indicates a percent change of zero, indicating no change in the number of cases from the previous year; above the line indicates more patients than the previous year and below the line fewer. B) Percent difference in prescriptions between 2019 and 2020 by month. NTSS case counts were aggregated by treatment start date month, whereas IQVIA data were aggregated by New to Brand prescription data for INH prescriptions, PZA prescriptions, or no filter applied. The horizontal black line represents no change in 2020 compared with 2019; above the line means more prescriptions in 2020 and below indicates fewer. The same analysis was also conducted for IQVIA projected patient counts (Appendix Figure 4). INH, isoniazid; NTSS, National Tuberculosis Surveillance System; PZA, pyrazinamide.

We next examined the percent difference each year during 2007–2020 (Figure 2, panel A). Using treatment start date to aggregate the data, we found that NTSS case counts decreased every year except 2007 and 2014. Before 2020, the largest decrease was in 2009, which coincided with the US economic recession (*15*), but a decrease of 26.7% occurred in 2020. This decrease is larger than reported previously (*2*), because treatment start date is missing for 2.8% of cases counted in 2019 and 5.2% of cases counted in 2020. Similarly, the isoniazid IQVIA projected patient counts generally decreased each year (except 2014 and 2016), and 2020 had the largest decrease (28.6%). In contrast, the pyrazinamide IQVIA patient counts revealed 4 years (2014, 2015, 2017, and 2019) in which more cases occurred than the previous year. Although 2020 pyrazinamide IQVIA patient counts had a large decrease (15.3%) compared with 2019, data for 2012 showed a larger decrease (17.2%), and the decrease in 2018 (14.4%) was similar. Azithromycin IQVIA data also showed multiple years with more projected patients than the previous year but a large drop in 2020 of −25.9% (Appendix Figure 1, panel C).

To further explore 2020 data, we compared each month of 2020 to the corresponding month in 2019 (Figure 2, panel B). For this analysis, we used NTB prescription data as the IQVIA metric because it has the strongest correlation after projected patient counts but enables users to look across all prescriptions. In NTSS data, approximately the same number of cases occurred in January and February 2020 as in January and February 2019 (i.e., the percent difference was near zero). However, starting in March, the percent difference dropped to −10% of 2019 values and stayed below that level for the rest of the year; the lowest percent difference (fewest patients in 2020 compared to 2019) occurred in April (−44%) and May (−42%). IQVIA isoniazid prescriptions followed a similar trend; the decrease began in March and never rose above −10% of 2019 values after March. For isoniazid, the April and May values were −50% and −52% of 2019 values. IQVIA pyrazinamide prescriptions in January and February were around 2019 levels, began to drop in March, and reached their lowest levels in April (−37.5%), May (−39.4%), and June (−35.5%) before increasing again. However, unlike NTSS and IQVIA isoniazid prescriptions, IQVIA pyrazinamide prescriptions came close to 2019 levels and even exceeded 2019 prescriptions in November 2020 before decreasing again in December. The overall IQVIA database (IQVIA no filter) also followed this pattern: there were more prescriptions in 2020 than in 2019 in January and February, a large drop in April and May, and another drop in December. We observed similar trends when using projected patient counts (Appendix Figure 4), although the decreases tended to be larger. Azithromycin prescriptions also largely followed this trend, except the lowest percent difference was in May (Appendix Figure 1, panel D).

###  Fewer IQVIA Projected Patient Counts in 2020, Similarity to NTSS Trend 

**Figure 3:**
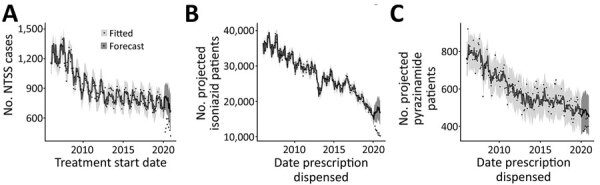
Tuberculosis cases reported to the National TB Surveillance System (NTSS) and IQVIA (https://www.iqvia.com) projected patient counts in 2020 compared with previous years, United States. A seasonal autoregressive integrated moving average model was fit to January 2006–December 2019 data from cases reported to NTSS by treatment start date (A), IQVIA isoniazid projected patient counts (B), or IQVIA pyrazinamide projected patient counts (C) (for model details, see Appendix Figures 6–8). Light gray indicates model with 95% prediction intervals, which was used to forecast 2020 counts with 95% prediction intervals (dark gray). Black dots represent the number of cases (A) or projected patient counts (B, C) each month. Vertical axes in each plot are different because of different scales.

We next analyzed whether the large declines in 2020 were within the error of what was expected on the basis of previous trends. NTSS case counts and IQVIA isoniazid and pyrazinamide projected patient counts all demonstrated a general downward trend, with seasonal effects causing regular fluctuations (Appendix Figure 5). Thus, we fit a SARIMA model to each metric (Figure 3). Overall, these models had a good fit, with most of the fitted data within the 95% prediction interval (Appendix Figures 6–8). As we found for the percent differences by month, the April–December 2020 NTSS monthly case counts and IQVIA isoniazid projected patient counts were all below the lower bound of the 95% prediction interval. In contrast, the IQVIA projected patient counts for pyrazinamide, although lower than the SARIMA model predictions for April–December, were only lower than the 95% prediction interval in April.

**Figure 4:**
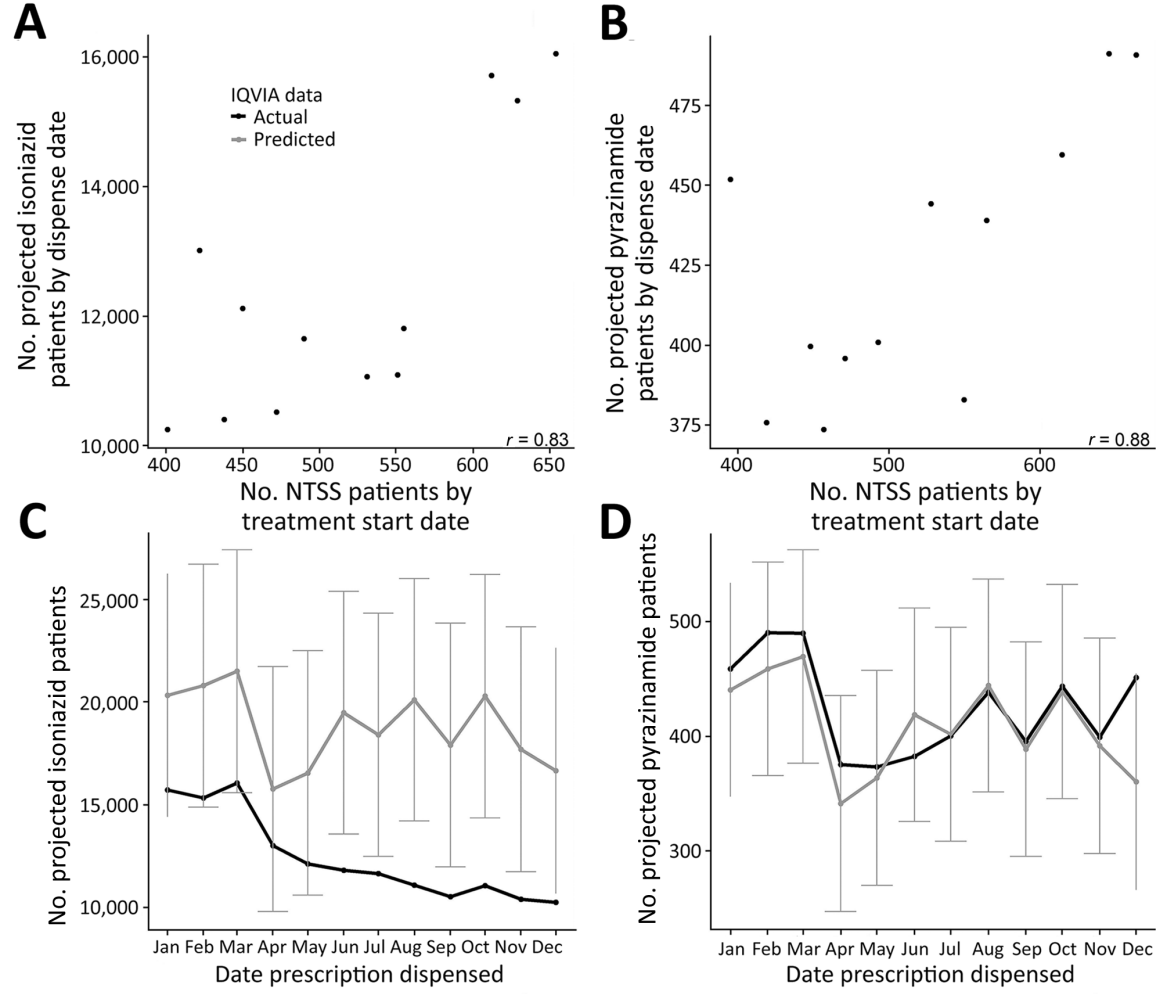
Comparison of National Tuberculosis Surveillance System (NTSS) case counts and IQVIA projected patient counts for isoniazid or pyrazinamide prescriptions, United States, 2020. A, B) Projected patient counts for isoniazid (A) and pyrazinamide (B). Horizontal axis of each plot shows NTSS patient counts aggregated by treatment start date (month), removing patients who had reported resistance. Each point represents a month in 2020. The Pearson correlation coefficient (*r*) is shown in lower righthand corner of each plot. C, D) A linear model fit to the 2006–2019 data (Figure 1) with quarter as a covariate to predict 2020 IQVIA projected patient counts for isoniazid (C) or pyrazinamide (D). Black line indicates actual data; gray line indicates expected IQVIA counts with 95% prediction intervals. Note vertical axes are different because of different scales for isoniazid and pyrazinamide in the IQVIA dataset. NTSS, National Tuberculosis Surveillance System.

We also explored whether the 2020 IQVIA data were within what would be expected from NTSS case counts. As we found for pre-2020 data, we observed a strong correlation (*r*≥0.83) between the NTSS patients counts and the IQVIA projected patient counts in 2020 (Figure 4, panels A, B). We used a linear model between the 2 datasets to predict IQVIA 2020 counts from NTSS 2020 counts. If the actual IQVIA counts were outside the prediction interval, this result would have been a sign that although both datasets decreased, other factors could be causing the decline to differ between the 2 datasets. We found that the predictions for the IQVIA isoniazid projected patient counts were lower than predicted for all months in 2020 and below the 95% prediction interval for June–December (Figure 4, panel C). Conversely, the IQVIA pyrazinamide projected patient counts were generally very close to prediction and were within the 95% prediction interval for all months (Figure 4, panel D).

## Discussion

Both NTSS case counts and IQVIA isoniazid, pyrazinamide, and azithromycin projected patient counts had a large decrease in 2020 compared with 2019; the lowest drops occurred in April and May. These months correspond to when large parts of the country had community mitigation measures in place, which resulted in decreased mobility, including delaying or avoiding of medical care (*19*,*20*). Although the percent change in the following months increased compared with values for April and May, changes still tended to be negative, suggesting that persons were not compensating by obtaining these prescriptions later in the year. In fact, based on our SARIMA models, the April–December 2020 declines were lower than expected from previous declining trends.

We found a strong correlation at the national level between NTSS case counts and isoniazid and pyrazinamide IQVIA projected patient counts, suggesting that isoniazid and pyrazinamide prescription data can serve as a proxy for trends in TB cases. In contrast, NTSS case counts and azithromycin (an antibiotic often used to treat chest infections such as pneumonia but not used for TB treatment) had a negative correlation, smaller in magnitude than isoniazid and pyrazinamide correlations. This finding suggests that this relationship is not being driven by general antibiotic prescriptions for respiratory diseases and supports the idea that the isoniazid and pyrazinamide relationship is specific to TB. In contrast, pyrazinamide, isoniazid, and azithromycin all had a strong state correlation, suggesting that the state relationship might be driven more by population than by the number of TB cases.

Differences between isoniazid and pyrazinamide prescriptions might be explained by the fact that isoniazid is prescribed for both LTBI and TB disease while pyrazinamide is prescribed only for TB disease and is generally used for only 2 months of disease treatment (as opposed to 6 months for isoniazid). For example, 2020 pyrazinamide counts were within prediction intervals from 2020 NTSS counts, whereas isoniazid counts were lower. This finding hints at a decline in LTBI treatment as well. However, the large decrease in 2020 compared with 2019, especially in April and May, was not specific to isoniazid and pyrazinamide; it was also seen in the overall database and in azithromycin prescriptions, indicating a general decline in the number of prescriptions dispensed in 2020.

The first limitation of our analysis is that IQVIA data only cover outpatient prescriptions, meaning that they are missing hospital prescriptions and are likely also missing prescriptions from health departments, which is where many TB patients receive their treatment. The proportion of TB cases covered by IQVIA pharmacy data is unknown, but total pyrazinamide TPT counts averaged 36% of NTSS case counts annually in this analysis. However, estimating the number of TB cases from pyrazinamide prescriptions is likely to be inaccurate, given that not all TB patients receive pyrazinamide treatment, doctors may prescribe pyrazinamide for off-label reasons, and treatments spanning multiple years would result in patients being counted twice. Given this limitation, we chose to focus on the trends between datasets. Despite the lack of hospital and health department data, we still found a strong correlation, suggesting this limitation does not affect the trends between datasets and that the proportion of TB cases in the IQVIA database has remained relatively consistent. Second, we did not analyze concurrent prescriptions (i.e., multiple drugs prescribed at the same time), which could change the results by helping separate TB disease and LTBI treatment. However, IQVIA data indicate only whether patients were prescribed multiple prescriptions at any point during the same year, rather than concurrently prescribed or initiated. Finally, a patient starting treatment at the start or end of the month might be counted in different months in the 2 databases.

If the IQVIA data had followed the NTSS trend for 2006–2019 but had not followed the NTSS trend for 2020, that finding would have suggested the decline in cases reported to NTSS was a surveillance artifact. However, IQVIA outpatient prescription data correlated with NTSS case counts from public health, which suggests that substantial underreporting is not a likely explanation for the decline in TB incidence in 2020. Even so, these analyses are not able to distinguish between a decline in actual TB incidence versus widespread underdiagnosis of TB (because of misdiagnosis as COVID-19 or because of persons with TB symptoms avoiding seeking medical attention out of fear of being exposed to or diagnosed with COVID-19). The COVID-19 pandemic has caused many disruptions to healthcare and public health, and delays in TB diagnosis because of the COVID-19 pandemic have already been reported (*21*). In fact, in the IQVIA data, the decrease was not specific to anti-TB medications but was seen when analyzing all prescriptions as well as azithromycin, even though azithromycin was not correlated with NTSS cases before 2019. Other studies have also reported large decreases in antibiotic prescriptions (*22*,*23*). Combined, this indicates a general overall decline in 2020 in prescriptions and, therefore, diagnoses.

This general decline is further supported by a study that reported that 41% of US adults had delayed or avoided seeking medical care by June 2020 (*20*). An analysis of health insurance claims from US persons with employer-sponsored insurance also showed large reductions (>20% for most services analyzed) in preventive and elective care, as well as in patient visits in March and April 2020 (*24*). In contrast, that study found prescription drug use for statins and antidiabetic medications decreased only 2%–3%, whereas asthma medication increased by 11%, although larger decreases in antibiotic prescriptions have been reported elsewhere (*22*,*23*). Thus, although healthcare use might have declined overall, this decline is not uniform across diseases. In fact, in the United States, the estimated number of newly diagnosed cancer cases in 2020 increased compared with 2019, but the number of HIV diagnoses decreased (31,670 in 2019 compared with 29,744 in 2020, a 6.1% decrease) (*25*–*28*). Thus, the decline in US TB cases and prescriptions was on par with declines in preventive care usage but was larger than the case counts and prescriptions for other diseases that result in longer treatments, although 2020 estimates for many conditions have not yet been reported. Nonetheless, TB cases should continue to be monitored closely, especially given the highly infectious nature of TB and the chance of increased illness and deaths if treatment is delayed.

Our study demonstrates that IQVIA isoniazid and pyrazinamide projected patient counts are strongly correlated with NTSS TB case counts and also shows large declines in 2020, helping to rule out underreporting as the cause of the large decline in reported TB cases in the United States. Evidence of underreporting would have suggested that public health practitioners were not aware of these cases but patients were still receiving timely treatment, resulting in decreased illness, deaths, and infectiousness. However, although we have provided evidence against underreporting, the strong possibility of underdiagnosis means that public health programs should be prepared for a possible rebound in TB cases after the pandemic, because delayed and missed diagnoses could result in increased transmission as patients remain infectious for longer periods of time. 

Appendix Additional information about the decrease in tuberculosis cases during COVID-19 pandemic as reflected by outpatient pharmacy data, United States, 2020
