# Supplementary material for: Decrease in Tuberculosis Cases during COVID-19 Pandemic as Reflected by Outpatient Pharmacy Data, United States, 2020
Source: Emerg Infect Dis. 2022 Apr;28(4):820–7. doi: 10.3201/eid2804.212014 (PMC8962886; doi:10.3201/eid2804.212014)
Supplement: Appendix — Additional information about the decrease in tuberculosis cases during COVID-19 pandemic as reflected by outpatient pharmacy data, United States, 2020 [file 21-2014-Techapp-s1.pdf]

Article DOI: https://doi.org/10.3201/eid2804.212014

# Decrease in Tuberculosis Cases during COVID-19 Pandemic as Reflected by Outpatient Pharmacy Data, United States, 2020

## Appendix

## Supplemental Methods

### IQVIA Database Overview

National Prescription Audit (NPA) captures new and refill prescriptions. NPA Extended Insights supplies more granular prescription data. NPA New to Brand (NTB) contains data on the first time a patient uses a brand. NPA Regional provides prescriptions at the state level (all other databases are at the national level). For all NPA databases, the only filter used was to set the Combined Molecule to isoniazid (INH), pyrazinamide (PZA), or "azithromycin." In the "no filter" analyses, no filters were set. These filters subset the IQVIA data to all prescriptions that contain the identified molecule as the active ingredient. Appendix Table 1 lists the timeframe and metrics used for each module while Appendix Table 2 defines each metric.

The previous modules are at the prescription-level, while Total Patient Tracker (TPT) is at the patient-level. It eliminates duplicate patients and multiple prescription fills to generate a final projected patient count. TPT data start in January 2002, but our analyses started in January 2006 because of the unreliability of data before then. TPT data were generated by creating a study that searched for INH or PZA as the only molecule and then generating a molecule report that excluded prescriptions from veterinarian specialties.

### Percent Change Calculation

Percent changes were used for general trends and were calculated as:

$$\frac{number\ of\ cases\ at\ time_i - number\ of\ cases\ at\ time_{i-1}}{number\ of\ cases\ at\ time_{i-1}} * 100$$

where $time_i$ indicates the year or month of interest and $time_{i-1}$ indicates the preceding year or month.

### Linear Models

For each drug, we tested 3 different linear models: no other covariates, month as a covariate, and quarter as a covariate. For both INH and PZA, the model with quarter had the best fit (as measured by lowest Akaike Information Criterion (*1*) and highest $R^2$), although all models had similar fits (data not shown). We therefore fit a linear model between IQVIA and NTSS data, 2006–2019, with quarter as categorical covariate:

$$IQVIA = \beta_0 + \beta_1 NTSS + \beta_2 quarter + \varepsilon$$

### References


1. Aikake H. A new look at the statistical model identification. IEEE Trans Automat Contr. 1974;19:716–23. https://doi.org/10.1109/TAC.1974.1100705

2. Hyndman RJ, Athanasopoulos G. Forecasting: principles and practice. Melbourne, Australia: OTexts; 2018 [cited 2021 Jul 27]. https://otexts.com/fpp2


**Appendix Table 1.** Overview of IQVIA prescription modules used to examine decrease in tuberculosis cases during COVID-19 pandemic, United States, 2020*

| Module | Earliest prescription data used | Metrics |
|---|---|---|
| NPA | February 2015 | NRx, RRx,TRx, EUNRx, EURRx, EUTRx |
| NPA Extended Insights | February 2018 | NRx, RRx,TRx, EUNRx, EURRx, EUTRx |
| NPA NTB | February 2015 | NRx, TRx, NBRx, NTS Rx, Cont Rx, Switch Add Rx |
| NPA Regional | February 2019 | NRx, RRx,TRx, EUNRx, EURRx, EUTRx |
| TPT | January 2006 | Projected patient count totals |

*Metrics are defined in Appendix Table 2. NPA, National Prescription Audit; NTB, New to Brand; TPT, Total Patient Tracker.

**Appendix Table 2.** Overview of IQVIA prescription metrics

| Metrics | Description |
|---|---|
| NRx | New prescription counts |
| RRx | Refill prescription counts |
| TRx | Total (new plus refill) prescription counts; TRx = NRx + RRx |
| EUNRx | Number of new dispensed units |
| EURRx | Number of refill dispensed units |
| EUTRx | Total number of dispensed units; EUTRx = EUNRx + EUTRx |
| NTS Rx | New therapy start prescription representing patients who have not used any therapy within the USC* market in the last 12 mo |
| Switch Add Rx | Switch/add prescriptions representing patients who in the past 12 mo have not used the brand previously but has used another drug in the USC* market in that time |
| NBRx | New to brand prescriptions representing the first-time use of a product within the previous 12 mo; NBRx = NTS Rx + Switch Add Rx |
| Cont Rx | Continuation on prescription representing repeat customers prescribed who have used the product in the past year |

*USC, uniform system of classification, developed by IQVIA to categorize products in same or similar markets.

**Appendix Table 3.** Correlations between NTSS and IQVIA isoniazid metrics*

| Timeframe | NTSS aggregation date | NTSS resistance inclusion | IQVIA database | IQVIA metric | No. timepoints compared | Pearson correlation coefficient |
|---|---|---|---|---|---|---|
| Month | Case date | No patients with drug resistance in NTSS | TPT | Projected patient count totals | 216 | 0.888 |
| Month | Treatment start date | No patients with drug resistance in NTSS | TPT | Projected patient count totals | 216 | 0.888 |
| Month | Treatment start date | All NTSS counts | TPT | Projected patient count totals | 216 | 0.885 |
| Month | Case date | All NTSS counts | TPT | Projected patient count totals | 216 | 0.854 |
| Quarter | Case date | No patients with drug resistance in NTSS | TPT | Projected patient count totals | 168 | 0.842 |
| Quarter | Treatment start date | No patients with drug resistance in NTSS | TPT | Projected patient count totals | 168 | 0.831 |
| Quarter | Treatment start date | All NTSS counts | TPT | Projected patient count totals | 168 | 0.825 |
| Quarter | Case date | All NTSS counts | TPT | Projected patient count totals | 168 | 0.782 |
| Month | Treatment start date | All NTSS counts | NPA NTB | NBRx | 59 | 0.644 |
| Month | Treatment start date | No patients with drug resistance in NTSS | NPA NTB | NBRx | 59 | 0.640 |
| Month | Treatment start date | All NTSS counts | NPA NTB | NTS Rx | 59 | 0.631 |
| Month | Treatment start date | No patients with drug resistance in NTSS | NPA NTB | NTS Rx | 59 | 0.630 |
| Month | Case date | All NTSS counts | NPA NTB | NBRx | 59 | 0.625 |
| Month | Case date | No patients with drug resistance in NTSS | NPA NTB | NBRx | 59 | 0.623 |
| Month | Case date | No patients with drug resistance in NTSS | NPA NTB | NTS Rx | 59 | 0.622 |
| Month | Case date | All NTSS counts | NPA Extended Insights | NRx | 23 | 0.621 |
| Month | Case date | All NTSS counts | NPA NTB | NTS Rx | 59 | 0.616 |
| Month | Case date | All NTSS counts | NPA Extended Insights | EUNRx | 23 | 0.610 |
| Month | Case date | No patients with drug resistance in NTSS | NPA NTB | NRx | 59 | 0.595 |
| Month | Case date | No patients with drug resistance in NTSS | NPA | NRx | 59 | 0.584 |
| Month | Case date | No patients with drug resistance in NTSS | NPA | EUNRx | 59 | 0.576 |
| Month | Treatment start date | No patients with drug resistance in NTSS | NPA NTB | NRx | 59 | 0.574 |
| Quarter | Treatment start date | No patients with drug resistance in NTSS | NPA | EUNRx | 20 | 0.566 |
| Month | Treatment start date | No patients with drug resistance in NTSS | NPA | NRx | 59 | 0.563 |
| Month | Treatment start date | All NTSS counts | NPA NTB | NRx | 59 | 0.554 |
| Month | Treatment start date | All NTSS counts | NPA | NRx | 59 | 0.541 |
| Quarter | Treatment start date | All NTSS counts | NPA | EUNRx | 20 | 0.539 |
| Month | Case date | All NTSS counts | NPA Extended Insights | TRx | 23 | 0.538 |
| Month | Case date | All NTSS counts | NPA Extended Insights | EUTRx | 23 | 0.526 |
| Month | Treatment start date | No patients with drug resistance in NTSS | NPA | EUNRx | 59 | 0.518 |
| Quarter | Treatment start date | All NTSS counts | NPA NTB | NTS Rx | 20 | 0.518 |

| Timeframe | NTSS aggregation date | NTSS resistance inclusion | IQVIA database | IQVIA metric | No. timepoints compared | Pearson correlation coefficient |
|---|---|---|---|---|---|---|
| Quarter | Treatment start date | All NTSS counts | NPA NTB | NBRx | 20 | 0.513 |
| Month | Case date | All NTSS counts | NPA NTB | NRx | 59 | 0.511 |
| Quarter | Treatment start date | No patients with drug resistance in NTSS | NPA NTB | NRx | 20 | 0.509 |
| Quarter | Treatment start date | No patients with drug resistance in NTSS | NPA NTB | NTS Rx | 20 | 0.508 |
| Month | Case date | No patients with drug resistance in NTSS | NPA Extended Insights | NRx | 23 | 0.501 |
| Quarter | Treatment start date | No patients with drug resistance in NTSS | NPA NTB | NBRx | 20 | 0.499 |
| Quarter | Treatment start date | No patients with drug resistance in NTSS | NPA | NRx | 20 | 0.498 |
| Month | Treatment start date | No patients with drug resistance in NTSS | NPA Extended Insights | NRx | 23 | 0.497 |
| Quarter | Treatment start date | All NTSS counts | NPA NTB | NRx | 20 | 0.496 |
| Month | Treatment start date | All NTSS counts | NPA | EUNRx | 59 | 0.496 |
| Month | Treatment start date | All NTSS counts | NPA Extended Insights | EUNRx | 23 | 0.494 |
| Month | Case date | No patients with drug resistance in NTSS | NPA Extended Insights | EUNRx | 23 | 0.494 |
| Month | Case date | All NTSS counts | NPA | NRx | 59 | 0.494 |
| Month | Treatment start date | All NTSS counts | NPA Extended Insights | NRx | 23 | 0.492 |
| Quarter | Treatment start date | No patients with drug resistance in NTSS | NPA | EUTRx | 20 | 0.489 |
| Month | Treatment start date | No patients with drug resistance in NTSS | NPA Extended Insights | EUNRx | 23 | 0.488 |
| Quarter | Treatment start date | All NTSS counts | NPA | NRx | 20 | 0.484 |
| Month | Case date | No patients with drug resistance in NTSS | NPA | EUTRx | 59 | 0.481 |
| Quarter | Case date | No patients with drug resistance in NTSS | NPA | EUNRx | 20 | 0.464 |
| Month | Treatment start date | No patients with drug resistance in NTSS | NPA NTB | TRx | 59 | 0.464 |
| Month | Case date | All NTSS counts | NPA | EUNRx | 59 | 0.462 |
| Month | Treatment start date | No patients with drug resistance in NTSS | NPA | TRx | 59 | 0.460 |
| Month | Case date | No patients with drug resistance in NTSS | NPA | TRx | 59 | 0.459 |
| Quarter | Treatment start date | All NTSS counts | NPA | EUTRx | 20 | 0.458 |
| Quarter | Case date | All NTSS counts | NPA NTB | NTS Rx | 20 | 0.458 |
| Month | Case date | No patients with drug resistance in NTSS | NPA NTB | TRx | 59 | 0.457 |
| Quarter | Case date | All NTSS counts | NPA NTB | NBRx | 20 | 0.450 |
| Month | Treatment start date | No patients with drug resistance in NTSS | NPA | EUTRx | 59 | 0.441 |
| Month | Treatment start date | All NTSS counts | NPA NTB | TRx | 59 | 0.440 |
| Month | Treatment start date | All NTSS counts | NPA | TRx | 59 | 0.435 |
| Quarter | Case date | All NTSS counts | NPA | EUNRx | 20 | 0.434 |
| Month | Case date | All NTSS counts | NPA Extended Insights | RRx | 23 | 0.420 |
| Month | Treatment start date | All NTSS counts | NPA | EUTRx | 59 | 0.415 |

| Timeframe | NTSS aggregation date | NTSS resistance inclusion | IQVIA database | IQVIA metric | No. timepoints compared | Pearson correlation coefficient |
|---|---|---|---|---|---|---|
| Month | Treatment start date | No patients with drug resistance in NTSS | NPA Extended Insights | TRx | 23 | 0.413 |
| Month | Treatment start date | All NTSS counts | NPA Extended Insights | EUTRx | 23 | 0.408 |
| Month | Case date | All NTSS counts | NPA Extended Insights | EURRx | 23 | 0.408 |
| Month | Treatment start date | No patients with drug resistance in NTSS | NPA Extended Insights | EUTRx | 23 | 0.407 |
| Quarter | Treatment start date | No patients with drug resistance in NTSS | NPA | EURRx | 20 | 0.401 |
| Month | Case date | No patients with drug resistance in NTSS | NPA Extended Insights | TRx | 23 | 0.401 |
| Month | Treatment start date | All NTSS counts | NPA Extended Insights | TRx | 23 | 0.400 |
| Quarter | Case date | All NTSS counts | NPA NTB | NRx | 20 | 0.399 |
| Month | Case date | No patients with drug resistance in NTSS | NPA Extended Insights | EUTRx | 23 | 0.398 |
| Quarter | Treatment start date | No patients with drug resistance in NTSS | NPA | TRx | 20 | 0.395 |
| Quarter | Treatment start date | No patients with drug resistance in NTSS | NPA NTB | TRx | 20 | 0.389 |
| Quarter | Case date | All NTSS counts | NPA | NRx | 20 | 0.383 |
| Month | Case date | All NTSS counts | NPA NTB | TRx | 59 | 0.382 |
| Quarter | Treatment start date | All NTSS counts | NPA | TRx | 20 | 0.377 |
| Month | Case date | All NTSS counts | NPA | TRx | 59 | 0.375 |
| Quarter | Treatment start date | All NTSS counts | NPA NTB | TRx | 20 | 0.373 |
| Month | Treatment start date | No patients with drug resistance in NTSS | NPA NTB | Cont Rx | 59 | 0.372 |
| Month | Case date | No patients with drug resistance in NTSS | NPA | EURRx | 59 | 0.370 |
| Month | Case date | No patients with drug resistance in NTSS | NPA NTB | Cont Rx | 59 | 0.370 |
| Quarter | Treatment start date | All NTSS counts | NPA | EURRx | 20 | 0.369 |
| Quarter | Case date | No patients with drug resistance in NTSS | NPA | EUTRx | 20 | 0.367 |
| Month | Case date | All NTSS counts | NPA | EUTRx | 59 | 0.359 |
| Quarter | Case date | No patients with drug resistance in NTSS | NPA NTB | NRx | 20 | 0.354 |
| Month | Treatment start date | No patients with drug resistance in NTSS | NPA | RRx | 59 | 0.351 |
| Month | Treatment start date | No patients with drug resistance in NTSS | NPA | EURRx | 59 | 0.349 |
| Quarter | Case date | No patients with drug resistance in NTSS | NPA | NRx | 20 | 0.346 |
| Quarter | Case date | No patients with drug resistance in NTSS | NPA NTB | NTS Rx | 20 | 0.345 |
| Month | Treatment start date | All NTSS counts | NPA NTB | Cont Rx | 59 | 0.342 |
| Quarter | Treatment start date | No patients with drug resistance in NTSS | NPA NTB | Cont Rx | 20 | 0.339 |
| Month | Case date | No patients with drug resistance in NTSS | NPA | RRx | 59 | 0.331 |
| Month | Treatment start date | All NTSS counts | NPA | RRx | 59 | 0.325 |
| Quarter | Case date | No patients with drug resistance in NTSS | NPA NTB | NBRx | 20 | 0.325 |
| Quarter | Case date | All NTSS counts | NPA | EUTRx | 20 | 0.324 |

| Timeframe | NTSS aggregation date | NTSS resistance inclusion | IQVIA database | IQVIA metric | No. timepoints compared | Pearson correlation coefficient |
|---|---|---|---|---|---|---|
| Month | Treatment start date | All NTSS counts | NPA | EURRx | 59 | 0.321 |
| Quarter | Treatment start date | All NTSS counts | NPA NTB | Cont Rx | 20 | 0.314 |
| Month | Treatment start date | No patients with drug resistance in NTSS | NPA Extended Insights | RRx | 23 | 0.303 |
| Month | Treatment start date | No patients with drug resistance in NTSS | NPA Extended Insights | EURRx | 23 | 0.300 |
| Quarter | Treatment start date | No patients with drug resistance in NTSS | NPA | RRx | 20 | 0.298 |
| Month | Treatment start date | All NTSS counts | NPA Extended Insights | EURRx | 23 | 0.297 |
| Month | Treatment start date | All NTSS counts | NPA Extended Insights | RRx | 23 | 0.283 |
| Quarter | Treatment start date | All NTSS counts | NPA | RRx | 20 | 0.278 |
| Month | Case date | No patients with drug resistance in NTSS | NPA Extended Insights | EURRx | 23 | 0.277 |
| Month | Case date | No patients with drug resistance in NTSS | NPA Extended Insights | RRx | 23 | 0.276 |
| Month | Case date | All NTSS counts | NPA NTB | Cont Rx | 59 | 0.275 |
| Quarter | Case date | All NTSS counts | NPA Extended Insights | EUNRx | 8 | 0.267 |
| Quarter | Case date | No patients with drug resistance in NTSS | NPA | EURRx | 20 | 0.265 |
| Quarter | Case date | All NTSS counts | NPA | TRx | 20 | 0.260 |
| Quarter | Case date | All NTSS counts | NPA NTB | TRx | 20 | 0.259 |
| Month | Case date | All NTSS counts | NPA | RRx | 59 | 0.256 |
| Month | Case date | All NTSS counts | NPA | EURRx | 59 | 0.247 |
| Quarter | Case date | No patients with drug resistance in NTSS | NPA | TRx | 20 | 0.216 |
| Quarter | Case date | All NTSS counts | NPA | EURRx | 20 | 0.212 |
| Quarter | Case date | No patients with drug resistance in NTSS | NPA NTB | TRx | 20 | 0.206 |
| Quarter | Case date | All NTSS counts | NPA NTB | Cont Rx | 20 | 0.190 |
| Quarter | Case date | All NTSS counts | NPA Extended Insights | EUTRx | 8 | 0.186 |
| Quarter | Case date | No patients with drug resistance in NTSS | NPA NTB | Cont Rx | 20 | 0.162 |
| Quarter | Case date | All NTSS counts | NPA | RRx | 20 | 0.152 |
| Quarter | Case date | All NTSS counts | NPA Extended Insights | NRx | 8 | 0.129 |
| Quarter | Case date | All NTSS counts | NPA Extended Insights | EURRx | 8 | 0.105 |
| Quarter | Case date | No patients with drug resistance in NTSS | NPA | RRx | 20 | 0.105 |
| Quarter | Case date | All NTSS counts | NPA Extended Insights | TRx | 8 | 0.048 |
| Quarter | Treatment start date | All NTSS counts | NPA Extended Insights | EUNRx | 8 | 0.030 |
| Quarter | Case date | No patients with drug resistance in NTSS | NPA Extended Insights | EUNRx | 8 | 0.000 |

| Timeframe | NTSS aggregation date | NTSS resistance inclusion | IQVIA database | IQVIA metric | No. timepoints compared | Pearson correlation coefficient |
|---|---|---|---|---|---|---|
| Quarter | Treatment start date | No patients with drug resistance in NTSS | NPA Extended Insights | EUNRx | 8 | −0.004 |
| Quarter | Case date | All NTSS counts | NPA Extended Insights | RRx | 8 | −0.015 |
| Month | Treatment start date | All NTSS counts | NPA NTB | Switch Add Rx | 59 | −0.038 |
| Quarter | Treatment start date | All NTSS counts | NPA Extended Insights | EUTRx | 8 | −0.052 |
| Month | Treatment start date | No patients with drug resistance in NTSS | NPA NTB | Switch Add Rx | 59 | −0.071 |
| Quarter | Treatment start date | No patients with drug resistance in NTSS | NPA Extended Insights | EUTRx | 8 | −0.081 |
| Quarter | Treatment start date | All NTSS counts | NPA Extended Insights | NRx | 8 | −0.085 |
| Quarter | Case date | No patients with drug resistance in NTSS | NPA Extended Insights | EUTRx | 8 | −0.092 |
| Month | Case date | All NTSS counts | NPA NTB | Switch Add Rx | 59 | −0.097 |
| Quarter | Treatment start date | No patients with drug resistance in NTSS | NPA Extended Insights | NRx | 8 | −0.113 |
| Quarter | Treatment start date | All NTSS counts | NPA Extended Insights | EURRx | 8 | −0.126 |
| Quarter | Case date | No patients with drug resistance in NTSS | NPA Extended Insights | NRx | 8 | −0.128 |
| Quarter | Treatment start date | No patients with drug resistance in NTSS | NPA Extended Insights | EURRx | 8 | −0.149 |
| Quarter | Treatment start date | All NTSS counts | NPA Extended Insights | TRx | 8 | −0.161 |
| Quarter | Case date | No patients with drug resistance in NTSS | NPA Extended Insights | EURRx | 8 | −0.174 |
| Quarter | Treatment start date | No patients with drug resistance in NTSS | NPA Extended Insights | TRx | 8 | −0.180 |
| Quarter | Case date | No patients with drug resistance in NTSS | NPA Extended Insights | TRx | 8 | −0.202 |
| Quarter | Treatment start date | All NTSS counts | NPA Extended Insights | RRx | 8 | −0.217 |
| Month | Case date | No patients with drug resistance in NTSS | NPA NTB | Switch Add Rx | 59 | −0.225 |
| Quarter | Treatment start date | No patients with drug resistance in NTSS | NPA Extended Insights | RRx | 8 | −0.229 |
| Quarter | Treatment start date | All NTSS counts | NPA NTB | Switch Add Rx | 20 | −0.242 |
| Quarter | Case date | No patients with drug resistance in NTSS | NPA Extended Insights | RRx | 8 | −0.257 |
| Quarter | Case date | All NTSS counts | NPA NTB | Switch Add Rx | 20 | −0.274 |
| Quarter | Treatment start date | No patients with drug resistance in NTSS | NPA NTB | Switch Add Rx | 20 | −0.296 |
| Quarter | Case date | No patients with drug resistance in NTSS | NPA NTB | Switch Add Rx | 20 | −0.435 |

*IQVIA database and metrics are described in Appendix Tables 1 and 2. NTSS, National Tuberculosis Surveillance System.

**Appendix Table 4.** Correlations between NTSS and IQVIA pyrazinamide metrics*

| Timeframe | NTSS aggregation date | NTSS resistance inclusion | IQVIA database | IQVIA metric | No. timepoints compared | Pearson correlation coefficient |
|---|---|---|---|---|---|---|
| Quarter | Treatment start date | No patients with drug resistance in NTSS | TPT | Projected Patient Count Totals | 168 | 0.879 |
| Quarter | Treatment start date | All NTSS counts | TPT | Projected Patient Count Totals | 168 | 0.874 |
| Month | Treatment start date | No patients with drug resistance in NTSS | TPT | Projected Patient Count Totals | 216 | 0.858 |
| Month | Treatment start date | All NTSS counts | TPT | Projected Patient Count Totals | 216 | 0.854 |
| Month | Case date | No patients with drug resistance in NTSS | TPT | Projected Patient Count Totals | 216 | 0.852 |
| Quarter | Case date | No patients with drug resistance in NTSS | TPT | Projected Patient Count Totals | 168 | 0.852 |
| Month | Case date | All NTSS counts | TPT | Projected Patient Count Totals | 216 | 0.843 |
| Quarter | Case date | All NTSS counts | TPT | Projected Patient Count Totals | 168 | 0.824 |
| Month | Treatment start date | No patients with drug resistance in NTSS | NPA NTB | NBRx | 59 | 0.658 |
| Month | Treatment start date | All NTSS counts | NPA NTB | NBRx | 59 | 0.651 |
| Month | Case date | No patients with drug resistance in NTSS | NPA NTB | NBRx | 59 | 0.605 |
| Month | Treatment start date | No patients with drug resistance in NTSS | NPA NTB | NTS Rx | 59 | 0.576 |
| Month | Treatment start date | No patients with drug resistance in NTSS | NPA | NRx | 59 | 0.574 |
| Month | Treatment start date | All NTSS counts | NPA NTB | NTS Rx | 59 | 0.565 |
| Month | Treatment start date | All NTSS counts | NPA | NRx | 59 | 0.559 |
| Month | Treatment start date | All NTSS counts | NPA NTB | NRx | 59 | 0.558 |
| Month | Treatment start date | No patients with drug resistance in NTSS | NPA NTB | NRx | 59 | 0.555 |
| Quarter | Treatment start date | No patients with drug resistance in NTSS | NPA NTB | NTS Rx | 20 | 0.549 |
| Month | Case date | No patients with drug resistance in NTSS | NPA NTB | NTS Rx | 59 | 0.545 |
| Quarter | Treatment start date | No patients with drug resistance in NTSS | NPA NTB | NBRx | 20 | 0.544 |
| Month | Treatment start date | No patients with drug resistance in NTSS | NPA | TRx | 59 | 0.520 |
| Month | Case date | No patients with drug resistance in NTSS | NPA | NRx | 59 | 0.518 |
| Month | Case date | All NTSS counts | NPA NTB | NBRx | 59 | 0.517 |
| Quarter | Treatment start date | All NTSS counts | NPA NTB | NTS Rx | 20 | 0.514 |
| Quarter | Treatment start date | All NTSS counts | NPA NTB | NBRx | 20 | 0.512 |
| Month | Treatment start date | No patients with drug resistance in NTSS | NPA NTB | TRx | 59 | 0.502 |
| Month | Case date | No patients with drug resistance in NTSS | NPA NTB | NRx | 59 | 0.502 |
| Month | Treatment start date | All NTSS counts | NPA NTB | TRx | 59 | 0.501 |
| Month | Treatment start date | All NTSS counts | NPA | TRx | 59 | 0.493 |
| Month | Treatment start date | All NTSS counts | NPA Extended Insights | NRx | 23 | 0.485 |
| Month | Case date | No patients with drug resistance in NTSS | NPA | TRx | 59 | 0.481 |
| Month | Case date | All NTSS counts | NPA NTB | NTS Rx | 59 | 0.476 |
| Month | Case date | No patients with drug resistance in NTSS | NPA NTB | TRx | 59 | 0.449 |

| Timeframe | NTSS aggregation date | NTSS resistance inclusion | IQVIA database | IQVIA metric | No. timepoints compared | Pearson correlation coefficient |
|---|---|---|---|---|---|---|
| Month | Treatment start date | All NTSS counts | NPA Extended Insights | EUNRx | 23 | 0.445 |
| Month | Treatment start date | All NTSS counts | NPA | EUNRx | 59 | 0.431 |
| Month | Treatment start date | No patients with drug resistance in NTSS | NPA | EUNRx | 59 | 0.430 |
| Month | Treatment start date | No patients with drug resistance in NTSS | NPA Extended Insights | NRx | 23 | 0.427 |
| Month | Case date | All NTSS counts | NPA NTB | NRx | 59 | 0.422 |
| Month | Treatment start date | No patients with drug resistance in NTSS | NPA Extended Insights | EUNRx | 23 | 0.407 |
| Month | Treatment start date | No patients with drug resistance in NTSS | NPA NTB | Switch Add Rx | 59 | 0.407 |
| Month | Treatment start date | All NTSS counts | NPA NTB | Switch Add Rx | 59 | 0.407 |
| Quarter | Treatment start date | No patients with drug resistance in NTSS | NPA NTB | Switch Add Rx | 20 | 0.401 |
| Quarter | Case date | All NTSS counts | NPA NTB | NTS Rx | 20 | 0.399 |
| Quarter | Treatment start date | No patients with drug resistance in NTSS | NPA | NRx | 20 | 0.388 |
| Month | Case date | All NTSS counts | NPA Extended Insights | EUNRx | 23 | 0.382 |
| Quarter | Treatment start date | All NTSS counts | NPA NTB | Switch Add Rx | 20 | 0.380 |
| Month | Case date | All NTSS counts | NPA | NRx | 59 | 0.380 |
| Month | Case date | All NTSS counts | NPA Extended Insights | NRx | 23 | 0.377 |
| Quarter | Case date | No patients with drug resistance in NTSS | NPA NTB | NTS Rx | 20 | 0.371 |
| Month | Treatment start date | No patients with drug resistance in NTSS | NPA | EUTRx | 59 | 0.370 |
| Month | Case date | No patients with drug resistance in NTSS | NPA | EUNRx | 59 | 0.369 |
| Month | Case date | No patients with drug resistance in NTSS | NPA Extended Insights | EUNRx | 23 | 0.360 |
| Month | Case date | No patients with drug resistance in NTSS | NPA NTB | Switch Add Rx | 59 | 0.359 |
| Month | Treatment start date | All NTSS counts | NPA | EUTRx | 59 | 0.356 |
| Quarter | Treatment start date | All NTSS counts | NPA | NRx | 20 | 0.356 |
| Quarter | Case date | All NTSS counts | NPA NTB | NBRx | 20 | 0.342 |
| Quarter | Treatment start date | No patients with drug resistance in NTSS | NPA NTB | NRx | 20 | 0.340 |
| Month | Treatment start date | All NTSS counts | NPA Extended Insights | TRx | 23 | 0.336 |
| Quarter | Treatment start date | All NTSS counts | NPA NTB | NRx | 20 | 0.331 |
| Month | Case date | No patients with drug resistance in NTSS | NPA | EUTRx | 59 | 0.331 |
| Quarter | Case date | No patients with drug resistance in NTSS | NPA NTB | NBRx | 20 | 0.329 |
| Month | Case date | All NTSS counts | NPA NTB | TRx | 59 | 0.326 |
| Quarter | Treatment start date | No patients with drug resistance in NTSS | NPA | TRx | 20 | 0.325 |
| Month | Case date | No patients with drug resistance in NTSS | NPA Extended Insights | NRx | 23 | 0.318 |
| Month | Case date | All NTSS counts | NPA | EUNRx | 59 | 0.305 |
| Month | Case date | All NTSS counts | NPA NTB | Switch Add Rx | 59 | 0.299 |

| Timeframe | NTSS aggregation date | NTSS resistance inclusion | IQVIA database | IQVIA metric | No. timepoints compared | Pearson correlation coefficient |
|---|---|---|---|---|---|---|
| Month | Case date | All NTSS counts | NPA | TRx | 59 | 0.295 |
| Month | Treatment start date | All NTSS counts | NPA Extended Insights | EUTRx | 23 | 0.292 |
| Month | Treatment start date | No patients with drug resistance in NTSS | NPA Extended Insights | TRx | 23 | 0.291 |
| Month | Case date | No patients with drug resistance in NTSS | NPA | RRx | 59 | 0.282 |
| Month | Treatment start date | No patients with drug resistance in NTSS | NPA | RRx | 59 | 0.281 |
| Quarter | Treatment start date | All NTSS counts | NPA | TRx | 20 | 0.281 |
| Month | Treatment start date | No patients with drug resistance in NTSS | NPA Extended Insights | EUTRx | 23 | 0.267 |
| Quarter | Treatment start date | No patients with drug resistance in NTSS | NPA NTB | TRx | 20 | 0.252 |
| Month | Treatment start date | All NTSS counts | NPA | RRx | 59 | 0.244 |
| Quarter | Treatment start date | No patients with drug resistance in NTSS | NPA | EUNRx | 20 | 0.236 |
| Quarter | Treatment start date | All NTSS counts | NPA NTB | TRx | 20 | 0.236 |
| Quarter | Treatment start date | All NTSS counts | NPA | EUNRx | 20 | 0.231 |
| Month | Case date | No patients with drug resistance in NTSS | NPA Extended Insights | EUTRx | 23 | 0.219 |
| Quarter | Case date | No patients with drug resistance in NTSS | NPA NTB | Switch Add Rx | 20 | 0.212 |
| Month | Case date | All NTSS counts | NPA Extended Insights | TRx | 23 | 0.212 |
| Quarter | Case date | All NTSS counts | NPA NTB | Switch Add Rx | 20 | 0.209 |
| Month | Case date | All NTSS counts | NPA | EUTRx | 59 | 0.200 |
| Quarter | Case date | All NTSS counts | NPA NTB | NRx | 20 | 0.191 |
| Month | Case date | No patients with drug resistance in NTSS | NPA Extended Insights | TRx | 23 | 0.185 |
| Month | Case date | All NTSS counts | NPA Extended Insights | EUTRx | 23 | 0.185 |
| Quarter | Case date | No patients with drug resistance in NTSS | NPA | NRx | 20 | 0.181 |
| Quarter | Case date | All NTSS counts | NPA | NRx | 20 | 0.173 |
| Quarter | Treatment start date | No patients with drug resistance in NTSS | NPA | RRx | 20 | 0.172 |
| Quarter | Treatment start date | No patients with drug resistance in NTSS | NPA | EUTRx | 20 | 0.160 |
| Quarter | Case date | No patients with drug resistance in NTSS | NPA | TRx | 20 | 0.150 |
| Month | Case date | No patients with drug resistance in NTSS | NPA | EURRx | 59 | 0.136 |
| Quarter | Case date | No patients with drug resistance in NTSS | NPA NTB | NRx | 20 | 0.135 |
| Quarter | Treatment start date | All NTSS counts | NPA | EUTRx | 20 | 0.135 |
| Month | Treatment start date | No patients with drug resistance in NTSS | NPA | EURRx | 59 | 0.121 |
| Quarter | Treatment start date | All NTSS counts | NPA | RRx | 20 | 0.112 |
| Quarter | Case date | All NTSS counts | NPA | EUNRx | 20 | 0.097 |
| Month | Treatment start date | All NTSS counts | NPA | EURRx | 59 | 0.088 |
| Quarter | Case date | All NTSS counts | NPA | TRx | 20 | 0.085 |
| Month | Case date | All NTSS counts | NPA | RRx | 59 | 0.076 |

| Timeframe | NTSS aggregation date | NTSS resistance inclusion | IQVIA database | IQVIA metric | No. timepoints compared | Pearson correlation coefficient |
|---|---|---|---|---|---|---|
| Quarter | Case date | No patients with drug resistance in NTSS | NPA | RRx | 20 | 0.076 |
| Quarter | Case date | All NTSS counts | NPA NTB | TRx | 20 | 0.070 |
| Month | Treatment start date | All NTSS counts | NPA NTB | Cont Rx | 59 | 0.064 |
| Month | Treatment start date | No patients with drug resistance in NTSS | NPA NTB | Cont Rx | 59 | 0.060 |
| Quarter | Case date | No patients with drug resistance in NTSS | NPA NTB | TRx | 20 | 0.055 |
| Quarter | Treatment start date | All NTSS counts | NPA Extended Insights | EUNRx | 8 | 0.054 |
| Quarter | Case date | No patients with drug resistance in NTSS | NPA | EUNRx | 20 | 0.040 |
| Month | Case date | No patients with drug resistance in NTSS | NPA NTB | Cont Rx | 59 | 0.036 |
| Quarter | Treatment start date | No patients with drug resistance in NTSS | NPA Extended Insights | EUNRx | 8 | 0.032 |
| Quarter | Treatment start date | All NTSS counts | NPA Extended Insights | NRx | 8 | 0.028 |
| Quarter | Case date | All NTSS counts | NPA Extended Insights | EUNRx | 8 | 0.022 |
| Quarter | Treatment start date | No patients with drug resistance in NTSS | NPA Extended Insights | NRx | 8 | 0.009 |
| Quarter | Case date | All NTSS counts | NPA Extended Insights | NRx | 8 | 0.006 |
| Quarter | Case date | No patients with drug resistance in NTSS | NPA | EUTRx | 20 | −0.001 |
| Quarter | Treatment start date | No patients with drug resistance in NTSS | NPA | EURRx | 20 | −0.003 |
| Quarter | Case date | All NTSS counts | NPA | EUTRx | 20 | −0.015 |
| Month | Case date | All NTSS counts | NPA NTB | Cont Rx | 59 | −0.049 |
| Month | Case date | All NTSS counts | NPA | EURRx | 59 | −0.059 |
| Quarter | Treatment start date | All NTSS counts | NPA | EURRx | 20 | −0.061 |
| Quarter | Treatment start date | All NTSS counts | NPA NTB | Cont Rx | 20 | −0.061 |
| Quarter | Treatment start date | No patients with drug resistance in NTSS | NPA NTB | Cont Rx | 20 | −0.062 |
| Quarter | Case date | No patients with drug resistance in NTSS | NPA | EURRx | 20 | −0.075 |
| Quarter | Case date | All NTSS counts | NPA | RRx | 20 | −0.079 |
| Quarter | Treatment start date | All NTSS counts | NPA Extended Insights | EUTRx | 8 | −0.081 |
| Month | Treatment start date | All NTSS counts | NPA Extended Insights | RRx | 23 | −0.084 |
| Month | Treatment start date | No patients with drug resistance in NTSS | NPA Extended Insights | RRx | 23 | −0.085 |
| Quarter | Treatment start date | No patients with drug resistance in NTSS | NPA Extended Insights | EUTRx | 8 | −0.090 |
| Quarter | Case date | No patients with drug resistance in NTSS | NPA Extended Insights | EUNRx | 8 | −0.091 |
| Month | Treatment start date | No patients with drug resistance in NTSS | NPA Extended Insights | EURRx | 23 | −0.103 |

| Timeframe | NTSS aggregation date | NTSS resistance inclusion | IQVIA database | IQVIA metric | No. timepoints compared | Pearson correlation coefficient |
|---|---|---|---|---|---|---|
| Month | Treatment start date | All NTSS counts | NPA Extended Insights | EURRx | 23 | −0.113 |
| Quarter | Treatment start date | All NTSS counts | NPA Extended Insights | TRx | 8 | −0.120 |
| Quarter | Case date | All NTSS counts | NPA Extended Insights | EUTRx | 8 | −0.129 |
| Month | Case date | No patients with drug resistance in NTSS | NPA Extended Insights | EURRx | 23 | −0.129 |
| Month | Case date | No patients with drug resistance in NTSS | NPA Extended Insights | RRx | 23 | −0.130 |
| Quarter | Treatment start date | No patients with drug resistance in NTSS | NPA Extended Insights | TRx | 8 | −0.133 |
| Quarter | Case date | All NTSS counts | NPA Extended Insights | TRx | 8 | −0.144 |
| Quarter | Case date | No patients with drug resistance in NTSS | NPA Extended Insights | NRx | 8 | −0.147 |
| Month | Case date | All NTSS counts | NPA Extended Insights | RRx | 23 | −0.170 |
| Quarter | Case date | All NTSS counts | NPA NTB | Cont Rx | 20 | −0.183 |
| Quarter | Case date | No patients with drug resistance in NTSS | NPA NTB | Cont Rx | 20 | −0.196 |
| Quarter | Case date | No patients with drug resistance in NTSS | NPA Extended Insights | EUTRx | 8 | −0.204 |
| Quarter | Case date | All NTSS counts | NPA | EURRx | 20 | −0.213 |
| Month | Case date | All NTSS counts | NPA Extended Insights | EURRx | 23 | −0.244 |
| Quarter | Case date | No patients with drug resistance in NTSS | NPA Extended Insights | TRx | 8 | −0.281 |
| Quarter | Treatment start date | No patients with drug resistance in NTSS | NPA Extended Insights | EURRx | 8 | −0.304 |
| Quarter | Treatment start date | All NTSS counts | NPA Extended Insights | EURRx | 8 | −0.318 |
| Quarter | Case date | No patients with drug resistance in NTSS | NPA Extended Insights | EURRx | 8 | −0.387 |
| Quarter | Case date | All NTSS counts | NPA Extended Insights | EURRx | 8 | −0.388 |
| Quarter | Treatment start date | No patients with drug resistance in NTSS | NPA Extended Insights | RRx | 8 | −0.388 |
| Quarter | Treatment start date | All NTSS counts | NPA Extended Insights | RRx | 8 | −0.389 |
| Quarter | Case date | All NTSS counts | NPA Extended Insights | RRx | 8 | −0.413 |
| Quarter | Case date | No patients with drug resistance in NTSS | NPA Extended Insights | RRx | 8 | −0.505 |

*IQVIA database and metrics are described in Appendix Tables 1 and 2. NTSS, National Tuberculosis Surveillance System.

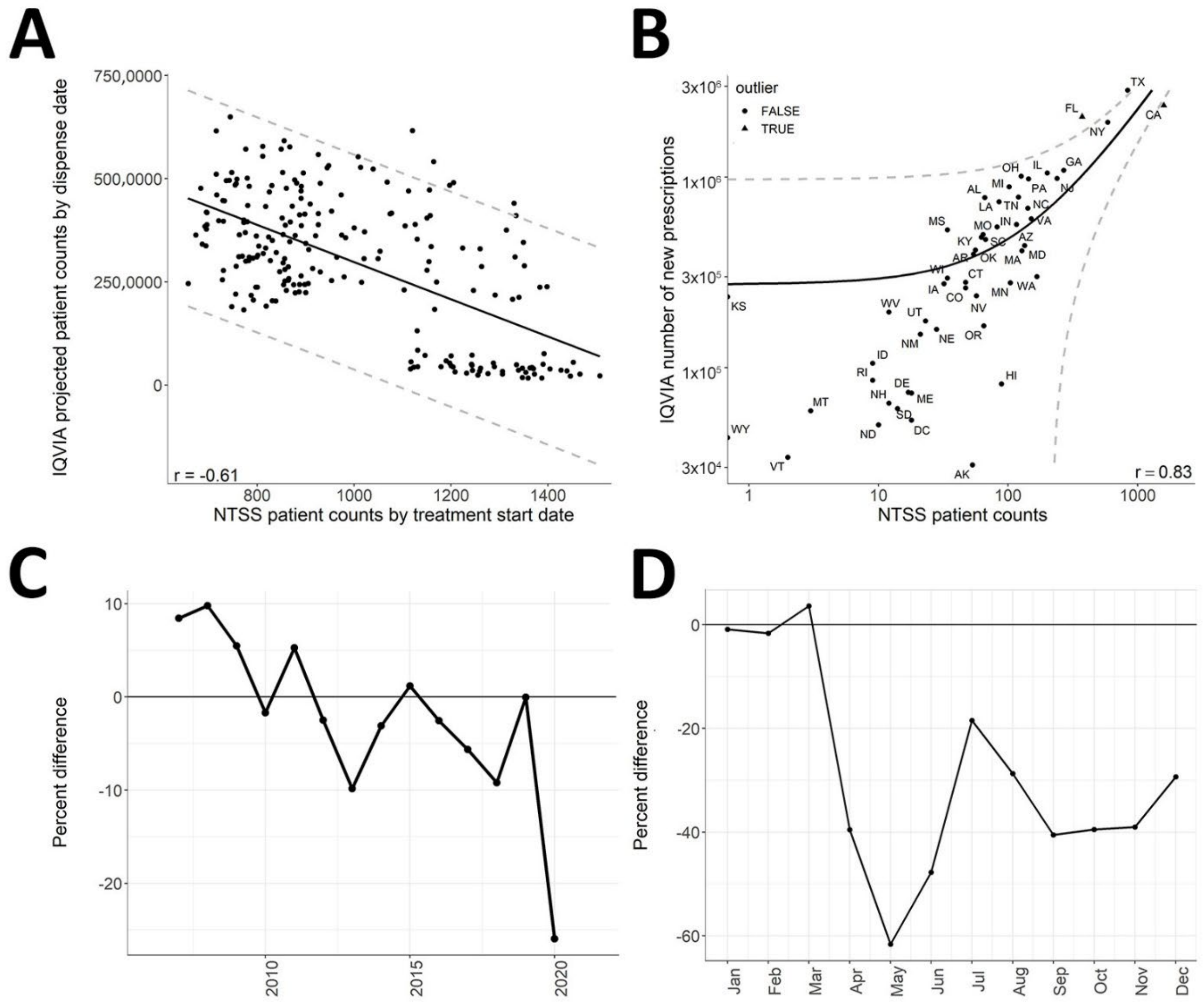


**Appendix Figure 1.** IQVIA azithromycin projected patient counts compared to NTSS data and changes over time. A) Horizontal axis of each plot is all NTSS patient counts aggregated by treatment start date month, while the vertical axis of each plot is the IQVIA azithromycin projected patient counts. Each point represents a month; all data at the national level during 2006–2019 is shown. B) Each point represents a state indicated by its 2-letter abbreviation. Triangles indicate outliers, defined as states with a standardized residual >3. Both axes are on a log scale. In panels A and B, the Pearson correlation coefficient (r) is shown in the corner of each plot, the solid black line represents a linear model between the 2 databases, and dashed gray lines indicate the 95% prediction interval. C) Annual percentage change compared to the previous year in IQVIA azithromycin projected patient counts. D) Monthly percent change comparing IQVIA azithromycin projected patient counts each month in 2020 to that month in 2019. NTSS, National Tuberculosis Surveillance System.

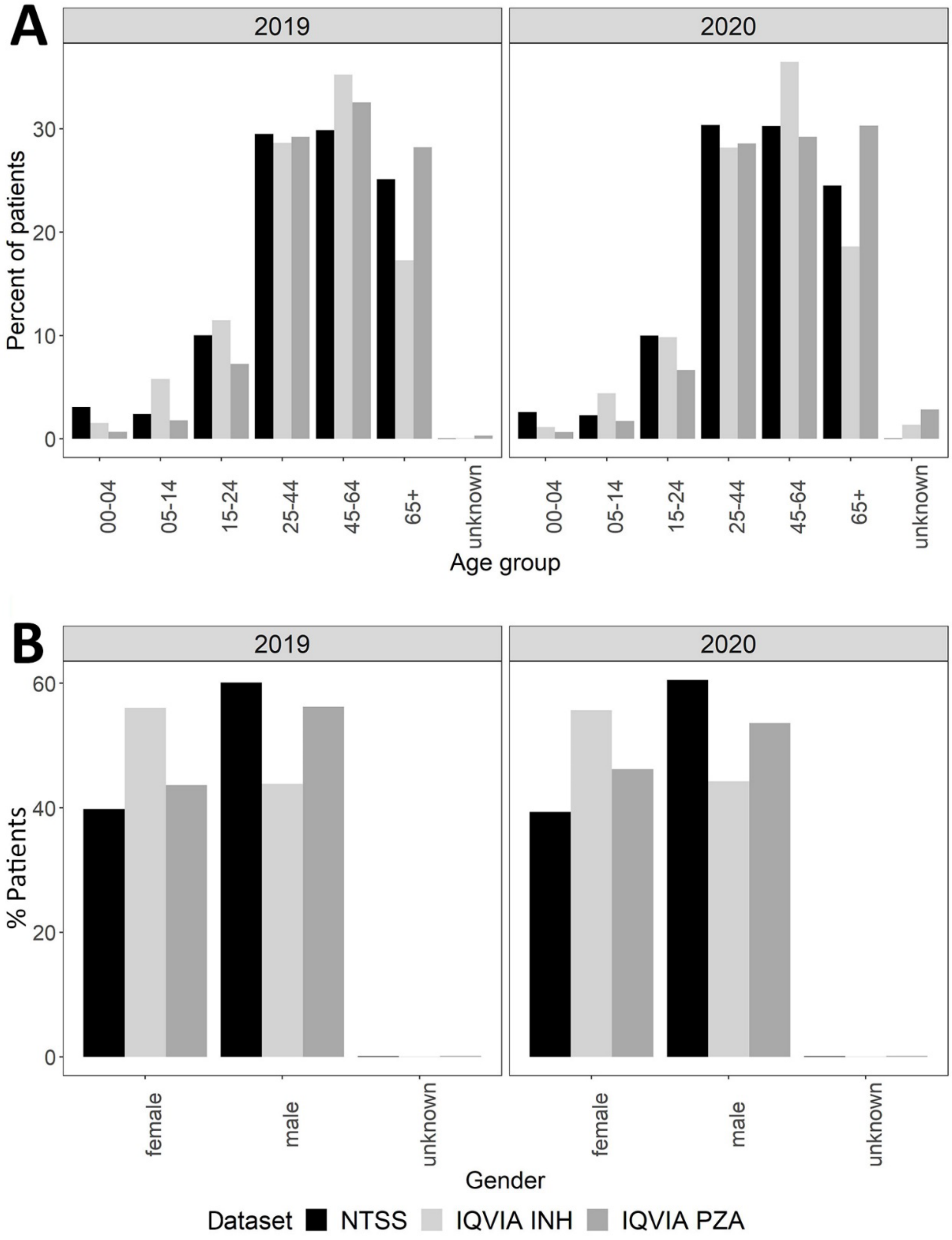


**Appendix Figure 2.** Comparison of patient demographics between the NTSS and IQVIA. A) Percentage of patients by age group for all NTSS cases (black), IQVIA INH projected patients (light gray), and IQVIA PZA projected patients (dark gray). Left plot shows the distribution in 2019 and right plot shows the distribution in 2020. B) Same as panel A except showing the percentage of patients by sex (NTSS) or gender (IQVIA). INH, isoniazid; NTSS, National Tuberculosis Surveillance System; PZA, pyrazinamide.

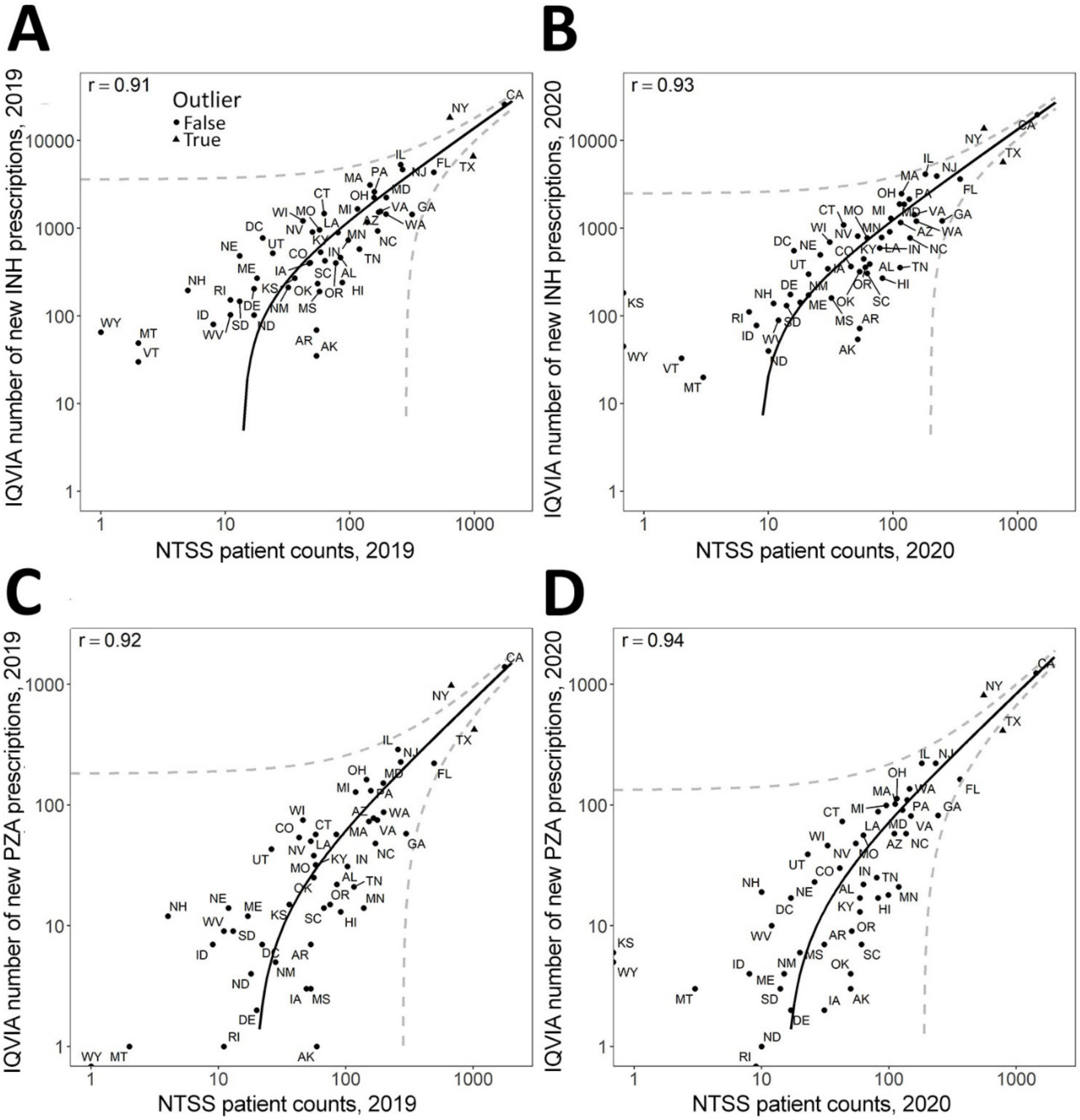


**Appendix Figure 3.** Comparison between National Tuberculosis Surveillance System (NTSS) and IQVIA data by state. The horizontal axis of each plot is the NTSS patient counts aggregated by treatment start date month and state, removing patients who had reported resistance to isoniazid (A–B) or pyrazinamide (C–D). On the vertical axis of each plot is the isoniazid (A–B) or pyrazinamide (C–D) IQVIA projected patient counts. Both axes are on a log scale. Panels A and C compare 2019 while panels B and D compare 2020 totals. IQVIA data are on different scales and so vertical axes are different. Each point represents a state indicated by its 2-letter abbreviation. Triangles indicate outliers, defined as states with a standardized residual >3. The Pearson correlation coefficient (r) is shown in the upper left-hand corner of each plot, solid black line represents a linear model between the 2 databases, and dashed gray lines indicate 95% prediction interval.

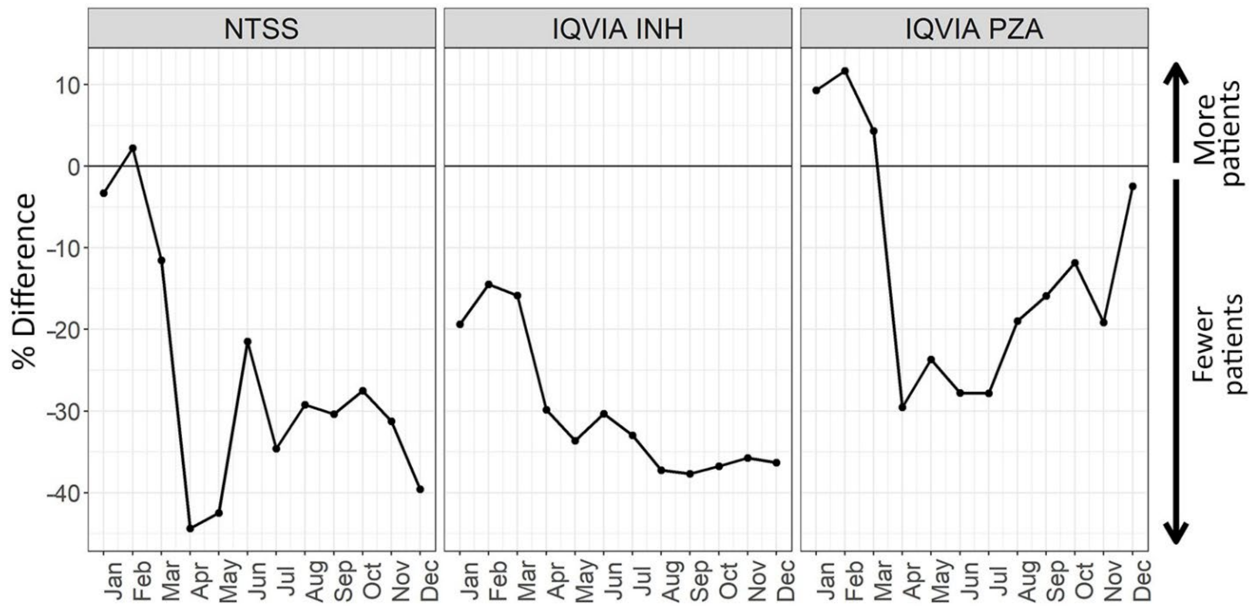


**Appendix Figure 4.** Percent difference in NTSS case counts and IQVIA projected patient counts between 2019 and 2020 by month. NTSS case counts were aggregated by treatment start date month while IQVIA data were aggregated by projected patient counts for INH or PZA. The horizontal black line represents no change in 2020 compared to 2019, above the line means more prescriptions in 2020, and below indicates less. INH, isoniazid; NTSS, National Tuberculosis Surveillance System; PZA, pyrazinamide.

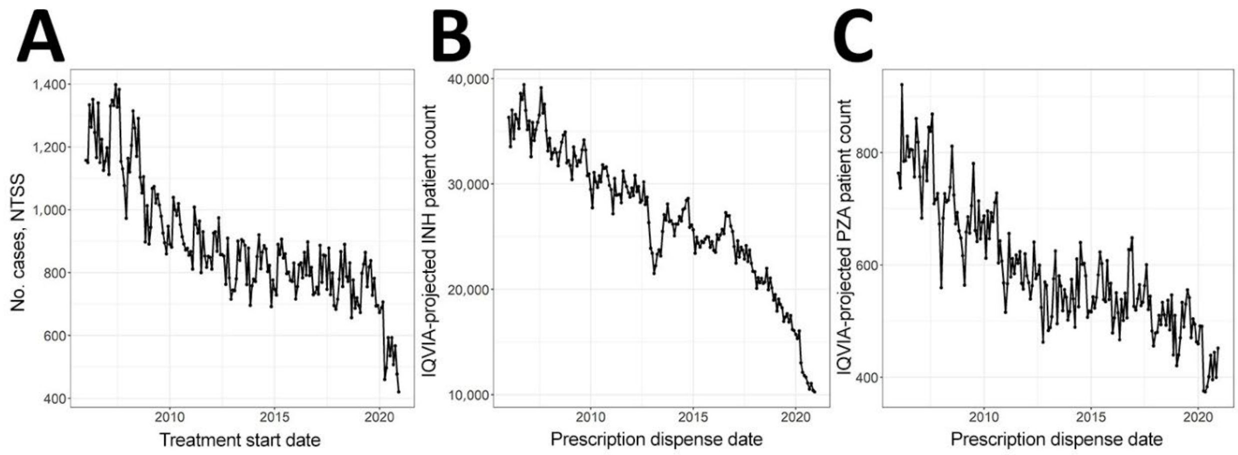


**Appendix Figure 5.** Database counts over time. Each point represents 1 month for A) NTSS cases aggregated by treatment start date, B) IQVIA INH projected patient counts by prescription dispense date, or C) IQVIA PZA projected patient counts by prescription dispense date. INH, isoniazid; NTSS, National Tuberculosis Surveillance System; PZA, pyrazinamide.

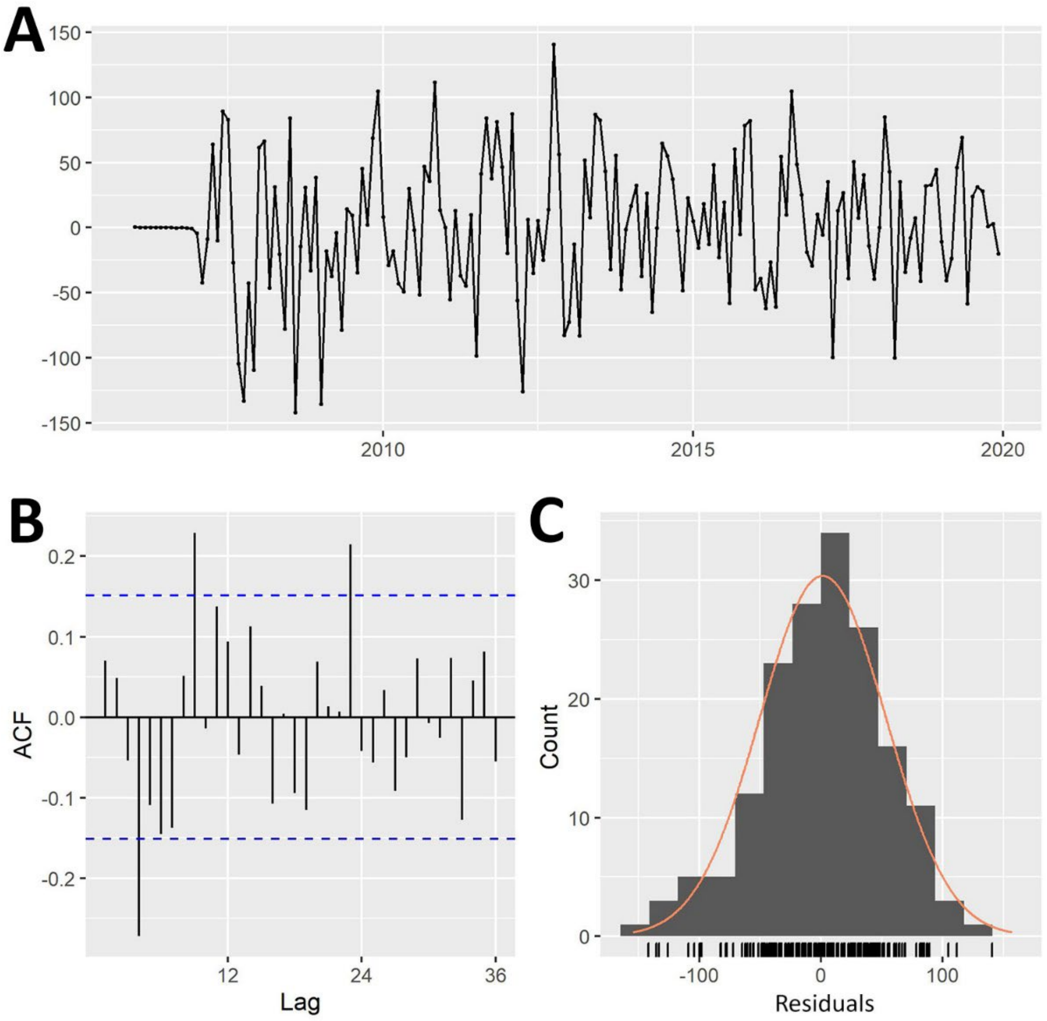


**Appendix Figure 6.** Residuals for the fitted National Tuberculosis Surveillance System seasonal ARIMA model. The specific model fitted is indicated in the title by ARIMA (p,d,q)(P,D,Q)[s], where (p,d,q) represents the non-seasonal part and (P,D,Q)[s] the seasonal part of the model (*2*). The number of autoregressive terms in each part are denoted by p and P, the degree of differencing by d and D, and the order of the moving averages by q and Q. The seasonal lag is denoted by s. In this instance, p = 2, d = D = Q = 1, q = P = 0, and s = 12 (indicating 12-month lags). ACF, autocorrelation function (represents the coefficients of correlation between a time series and lags of itself). ARIMA, autoregressive integrated moving average.

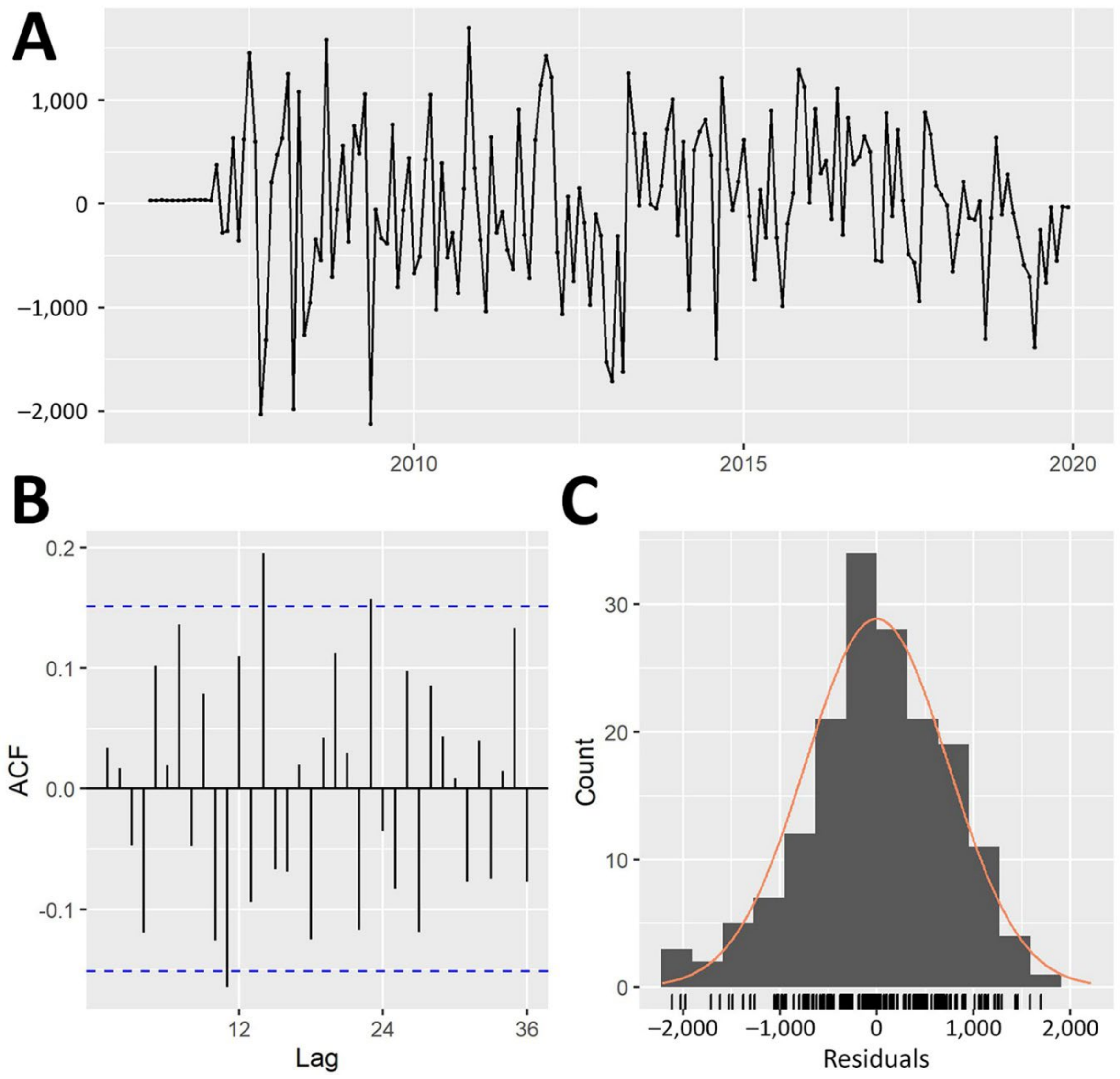


**Appendix Figure 7.** Residuals for the fitted IQVIA isoniazid SARIMA model. The specific model fitted is indicated in the title by ARIMA (p,d,q)(P,D,Q)[s], where (p,d,q) represents the non-seasonal part and (P,D,Q)[s] the seasonal part of the model (2). The number of autoregressive terms in each part are denoted by p and P, the degree of differencing by d and D, and the order of the moving averages by q and Q. The seasonal lag is denoted by s. In this instance, p = 4, d = q = P = 0, D = Q = 1, and s = 12 (indicating 12-month lags). ACF, autocorrelation function (represents the coefficients of correlation between a time series and lags of itself). ARIMA, autoregressive integrated moving average.

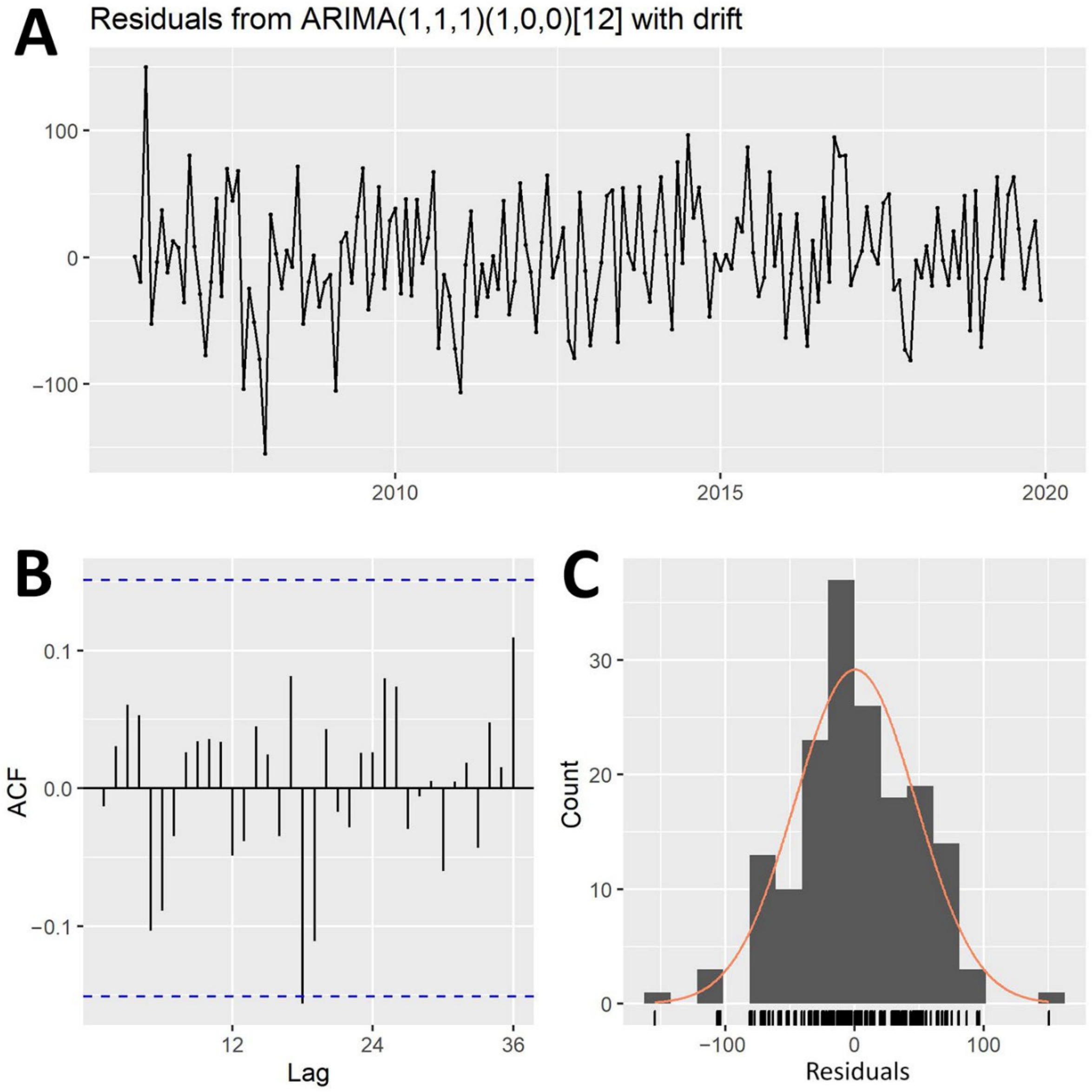


**Appendix Figure 8.** Residuals for the fitted IQVIA pyrazinamide SARIMA model. The specific model fitted is indicated in the title by ARIMA (p,d,q)(P,D,Q)[s], where (p,d,q) represents the non-seasonal part and (P,D,Q)[s] the seasonal part of the model (*2*). The number of autoregressive terms in each part are denoted by p and P, the degree of differencing by d and D, and the order of the moving averages by q and Q. The seasonal lag is denoted by s. In this instance, p = d = q = P = 1, D = Q = 0, and s = 12 (indicating 12-month lags). ACF, autocorrelation function (represents the coefficients of correlation between a time series and lags of itself). ARIMA, autoregressive integrated moving average.
